# Nutritional Content and Health Profile of Non-Dairy Plant-Based Yogurt Alternatives

**DOI:** 10.3390/nu13114069

**Published:** 2021-11-14

**Authors:** Winston J. Craig, Cecilia J. Brothers

**Affiliations:** 1 Center for Nutrition, Healthy Lifestyles, and Disease Prevention, School of Public Health, Loma Linda University, Loma Linda, CA 92354, USA; 2 Department of Biology, Walla Walla University, College Place, WA 99324, USA; cecilia.brothers@wallawalla.edu

**Keywords:** non-dairy yogurt alternatives, plant-based yogurts, nutrient composition, fortification, calcium, vitamin D, vitamin B12, protein, sugar

## Abstract

Yogurt is considered a healthy, nutritious food in many cultures. With a significant number of people experiencing dairy intolerance, and support for a more sustainable diet, consumer demand for dairy alternatives has surged. The aim of this study was to conduct a cross-sectional survey of plant-based yogurt alternatives to assess their nutritional content and health profile. A total of 249 non-dairy yogurt alternatives were analyzed from the nutrition label listed on the commercial package. The various yogurt alternatives contained extracts of coconut (*n* = 79), almonds (*n* = 62), other nuts or seeds (*n* = 20), oats (*n* = 20), legumes (*n* = 16), and mixed blends (*n* = 52). At least one-third of the yogurt alternatives had 5 g or more of protein/serving. Only 45% of the yogurt alternatives had calcium levels fortified to at least 10% of daily value (DV), while only about one in five had adequate vitamin D and B12 fortification at the 10% DV level. One-half of the yogurt alternatives had high sugar levels, while 93% were low in sodium. Except for the coconut-based products, the yogurts were not high in fat or saturated fat. The yogurt alternatives were not fortified as frequently or to the same levels as the corresponding non-dairy, plant-based beverages.

## 1. Introduction

Yogurt, plain or sweetened, is a very popular food in many cultures. In Europe, the dietary recommendations suggest the consumption of 100–250 g of yogurt/day [1]. Yogurt is considered a tasty, healthy and nutritious food, supplying some important vitamins and minerals. Individuals concerned about following a more sustainable diet, those with a dairy intolerance, or who desire a non-dairy alternative due to dietary preferences, such as vegans, will choose a lactose-free plant–based yogurt alternative. Typically, the comfort level of such individuals, and others who wish to experiment with new foods, is best met when the yogurt alternative has a similar appearance and texture to the dairy product. The growing interest in non-dairy yogurts, combined with the surge of interest in plant-based milk alternatives and meat alternatives has fueled a plant-based food industry valued at USD 5 billion [2] that is re-shaping the future of American cuisine. Over the past 15 years the number of Americans following plant-based diets has surged 300% [2]. The global plant-based yogurt market was valued at USD 1.6 billion in 2019 and is projected to grow at an annual growth rate of nearly 20% from 2020 to 2027 [3]. The US market for plant-based yogurt alternatives was about USD 400 million in 2020 and is expected to be valued at USD 1.3 billion by 2027 [3].

The popularity of yogurt alternatives over the past decade was especially prominent among millennials [3]. A number of manufacturers have made a decided effort to market their plant-based yogurt alternatives to a generation who take sustainability and the health of the planet seriously. Their websites clearly display that emphasis in their stated goals and mission [4,5,6]. During production of plant-based yogurt alternatives, companies claim they work for the health of the planet, conserve resources, and reduce the environmental impact and the carbon footprint of their activities. Some claim to use fair trade ingredients, and most claim to be vegan, GMO (genetically modified organisms)-free, and organic. The plant-based yogurts currently are based upon almonds, coconut, oats, or a legume (soy or yellow pea). With over 35 flavors available, there are many varieties from which to select. In addition, some plant-based yogurt alternatives possess unique health properties. For example, yogurts based on flax or hemp contain substantial levels of omega-3 fatty acids and fiber.

Consumers who regularly rely upon a non-dairy alternative may place themselves a little short nutritionally. If an appropriate substitution of a dairy product is not made, one could experience a nutritional shortfall in protein, calcium and some micronutrients. For a vegan, and someone who limits their intake of animal products, there are three nutrients of special concern. These are calcium, vitamin D, and vitamin B12 [7]. Calcium and vitamin B12 are supplied by dairy yogurt, while vitamin D is only supplied by those dairy yogurts fortified with vitamin D. On the other hand, these three nutrients are not supplied by plant-based yogurt alternatives unless the products are fortified. When plant-based non-dairy beverages are fortified, they may have similar amounts of calcium, vitamins D and B12 as dairy milk or they may be fortified with only one or two of the three critical nutrients [8,9]. We set out to see how many of the non-dairy yogurt alternatives have adequate protein levels and fortification, and at what level.

People choosing to follow a plant-based diet may need substantial guidance from a health care professional (such as a dietitian) to select a well-fortified non-dairy product. They may not know the nutritional pros and cons of consuming a particular non-dairy beverage versus a particular yogurt alternative. The choice should be influenced by which provides the better level of protein, calcium, vitamin D, and/or vitamin B12. They may prefer to consume a non-dairy beverage long-term rather than a yogurt alternative, or vice-versa, and would be interested to know how their nutritional status would be impacted by their preference. Hence, we set out to see how the nutrient levels in a yogurt alternative compared (on a serving basis) with those from a similar analysis of non-dairy plant-based beverages [8].

Consumers are normally concerned, for health reasons, about the level of sodium, sugar, and fat/saturated fat that exists in the food they purchase. We therefore examined the levels of these nutrients to see how many of the yogurt alternatives had acceptable levels of these nutrients. Furthermore, fermented foods are quite popular due to the perception that they are both nutritious and have health benefits from the bioactive metabolites produced by the fermentation [10,11]. Recently, the consumption of fermented foods containing live microorganisms has been seen as an important dietary approach for improving human health [12]. In addition, water-soluble dietary fibers, such as gums and inulin, are considered probiotic compounds, due to their fermentability by gut microbiota. These fibers provide a variety of health benefits, including an improved immune system defense [13,14]. We also examined the extent to which the plant-based yogurt alternatives contained prebiotics and probiotics.

## 2. Materials and Methods

The nutritional contents of 249 plant-based yogurt alternatives, representing 34 brands, were analyzed. The yogurts were selected, from March to May 2021, from those available in supermarkets and convenience stores in the western USA. Additional varieties of plant-based, non-dairy yogurt alternatives [15] were analyzed from the nutritional labels given by the manufacturer’s website, or from the website of common retailers. The plant-based yogurts with incomplete nutrition data were not included in the analysis.

The nutritional content of each yogurt was recorded from the nutrition label on the commercial package or from the information located on the website of the manufacturer or retailer. The nutrients per serving size, which were available on all packages, included calories, fat, saturated fat, sodium, carbohydrates, dietary fiber, total sugars, protein, and the micronutrients calcium, vitamin D, and vitamin B12. The median values of the nutrients were calculated for each type of yogurt.

A similar analysis was conducted on 326 multi-serve, plant-based, non-dairy beverages available in the USA, so that a comparison could be made between the nutritional value of one serving of the plant-based yogurt alternative with that of one serving of a plant-based beverage. The levels of fortification for calcium, vitamin D, and vitamin B12 were calculated for all yogurts and beverages separately.

The nutritional value of each plant-based yogurt was rated according to the following criterion: calcium, vitamin D and vitamin B12 of at least 10% of daily value (DV)/serving, and at least 5 g of protein/serving (10% of the DV). The health qualities demonstrated by the ingredients were determined by the following criteria: not more than 5 g of total sugars/serving; not more than 1 g of saturated fat/serving; not more than 150 calories/serving; not more than 115 mg sodium/serving; and at least 1.5 g of dietary fiber/serving. In addition, we noted how many yogurts had high levels of sugar, fat, and saturated fat. This was recorded as the number of yogurts having 10 g or more of total sugars/serving (20% DV for sugars), having more than 15.5 g fat/serving (20% DV for fat) and having 4 g or more of saturated fat/serving (20% DV for saturated fat). The US Dietary Guidelines specify, as a general guide, that 5% DV or less of a nutrient/serving is considered low, while 20% DV or more of a nutrient/serving is considered high [16,17]. In the USA, the DV for calcium is 1300 mg, vitamin D is 20 mcg, vitamin B12 is 2.4 mcg, sodium is 2300 mg, protein is 50 g, added sugars is 50 g, saturated fat is 20 g, and dietary fiber is 28 g [17]. For our analyses, we considered a 10% DV as an adequate fortification for calcium, vitamin D and vitamin B12. For sodium and saturated fat we accepted that beverages should not exceed 5% of their DV (a designated low level), namely 115 mg for sodium (5% of 2300 mg) and 1 g of saturated fat (5% of 20 g). We suggest that yogurts have at least 5% of DV for dietary fiber (approx. 1.5 g), at least 10% of the DV/serving for protein (5 g), and no more than 10% DV/serving for sugars. Ten percent was chosen as a mid-stream number between the 5% DV (low value) and the 20% DV (high value). This gave us a minimally acceptable level of 5 g protein/serving, and a level of 5 g sugars/serving.

### Statistical Analysis

R software was used to conduct all statistical analyses [18]. Data were tested for normality and homoscedasticity prior to analysis. The median and interquartile range were used for descriptive statistics, as the data were not normally distributed. The nutritional content was compared across the types of non-dairy yogurt bases using a Kruskal-Wallis test for each nutrient, followed by Dunn’s post hoc test with Bonferroni adjustment for multiple comparisons. The nutritional content was analyzed between non-dairy yogurt and beverage alternatives using an unpaired Wilcoxon rank sum test for each nutrient. An unpaired Wilcoxon rank sum test was also used to compare the levels of fortified nutrients between non-dairy yogurt and beverage alternatives for each fortified nutrient. The nutritional content was compared across product type (beverage or yogurt) and base type using a two-way analysis of variance for each nutrient, followed by Tukey’s honestly significant difference test. Only significant pairwise comparisons between non-dairy yogurt and beverage alternatives for the same base type were reported. A significant *p*-value of less than 0.05 was used for all analyses.

## 3. Results

The 249 plant-based yogurt alternatives analyzed were based upon coconut (*n* = 79), almonds (*n* = 62), oats (*n* = 20), cashews (*n* = 13), soy (*n* = 11), pea protein (*n* = 5), hemp (*n* = 4), pumpkin seed (*n* = 3), and the following mixtures: coconut with pea protein (*n* = 23), oats with pea protein and/or fava bean (*n* = 16), coconut with pili nut (*n* = 6), almond with fava bean (*n* = 4), coconut with cashews and watermelon and pumpkin seeds (*n* = 3). Table 1 displays the medians of each nutrient for all of these base types, and differences among the base types are reported. 

Two hundred and twelve (85.1%) of the yogurt alternatives were flavored while 24 (9.6%) were unsweetened and 36 were labeled as plain (14.5%). The most common flavors were vanilla (19%), strawberry (14%), and blueberry (10%). Twenty-three (68%) of the brands had no fortification. Only 11 of the 34 brands had calcium fortification. Tricalcium phosphate (TCP) (47%), calcium citrate (17%), and a TCP-calcium citrate mix (18%) were the most commonly used calcium salts. Others included calcium lactate-TCP mix (10%), and calcium carbonate-TCP mix (5%), and calcium carbonate (4%). At least two brands of yogurt alternatives used stevia and/or monk fruit extracts for sweetening. Added fibers included pectin (64% of brands), locust bean gum (45%), agar (12%) and guar (6%). Acacia gum and gellan were rarely used. Six of the 34 brands contained inulin from either chicory root or agave. Each yogurt typically contained 4–6 live cultures. Eight almond-based and three coconut-based yogurts contained mix-ins. All 11 with mix-ins contain 180 calories and above, with a mean of 217 calories/serving. One serving size of yogurt alternative varied according to the size of the container. The most common sizes encountered were 150 g (66.7%), 170 g (14.1%), and 120 g (7.6%).

Table 2 summarizes the data showing the percentage of the yogurt alternatives that contain a) a reasonable level of important and essential nutrients, b) low levels of sugar, sodium and saturated fat, and c) high levels of sugar, fat, saturated fat and calories. In Table 3, the data for the yogurt alternatives that meet or exceed suggested nutrient guidelines are separated out according to the different bases for comparison.

Table 4, Table 5 and Table 6 compare the nutrient composition of plant-based yogurt alternatives with plant-based beverages. Table 4 summarizes the nutrient composition of the yogurt alternatives and compares the medians for each nutrient with the medians of the nutrients in the non-dairy plant-based beverages. Significant differences were observed between yogurt and beverage alternatives for each nutrient. Table 5 reports the fortification levels of the yogurt alternatives and compares the levels of each of the three nutrients (calcium, vitamins D and B12) in the yogurt alternatives with the corresponding levels in the plant-based beverages. Significant differences were observed between yogurt and beverage alternatives for each fortified nutrient.

The 326 plant-based beverage alternatives analyzed were based upon almonds (*n* = 83), oats (*n* = 62), soy (*n* = 44), coconut (*n* = 24), hemp (*n* = 15), pea protein (*n* = 14), rice (*n* = 11), cashews (*n* = 10), flax (*n* = 6), banana (*n* = 6), macadamia (*n* = 6), hazelnuts (*n* = 4), chia (*n* = 4), quinoa (*n* = 2), pili nut (*n* = 2), walnut (*n* = 1), pistachio (*n* = 1), and the following mixtures: almond and pea (*n* = 7), almond and coconut (*n* = 5), sesame and pea (*n* = 5), flax and pea (*n* = 4), oats and pea (*n* = 2), oats and avocado (*n* = 2), rice and quinoa (*n* = 2), almond and cashew (*n* = 2), coconut, cashew, and oats (*n* = 1), and almond and sesame (*n* = 1; Table 6). For the plant-based beverage alternatives, 121 (37.1%) were unsweetened. While 60% of the beverage alternatives were plain, the most common flavors were vanilla (25%) and chocolate (9.5%). The typical serving size of the beverage was 240 mls.

## 4. Discussion

While non-dairy, plant-based yogurt alternatives are becoming more popular, concerns exist regarding their nutritional value relative to regular dairy yogurts. Of special concern is the level of protein, and the level of fortification of calcium, vitamin D and vitamin B12. The latter three nutrients are especially needed by a vegan and others consuming a plant-based diet that is devoid of adequate fortification. The median values of nutrients in the non-dairy yogurt alternatives, as reported for the overall total of 249 products in Table 1, are similar to those of a leading brand of 2% dairy yogurt (Table S1), except for the higher value of fiber, and lower protein and saturated fat content in the non-dairy alternatives. A recent analysis of dairy yogurts and non-dairy yogurt alternatives revealed that the levels of protein and calcium in the dairy yogurts were significantly higher than the yogurt alternatives (Table S2) [19]. One notes that the nutritional values for a Greek-style yogurt typically show considerably less calcium than a regular yogurt, while its protein content can be as high as 15–20 g/serving [20].

While the median value of protein in our non-dairy plant-based yogurt alternatives was only 3 g, all the legume-containing products (with soy, pea protein or fava bean) had a median of 6 g protein/serving while those based on seeds had a median of 8 g/serving. In fact, one-third (32.5%) of the non-dairy yogurt alternatives had at least 5 g of protein per serving, with 29% of the products containing 5 to 8 g protein/serving, and 4% of the yogurts contained as much as 10–11 g protein/serving. The overall picture for protein (a mean value of only 3 g/serving) was impacted considerably by coconut-based products which accounted for one-third of the yogurt alternatives and had a median value of only 1 g protein/serving. For comparison, the median protein level of 38 non-dairy plant-based yogurt alternatives, in a UK study, was 3.6 g/100 g (equivalent to 5.4 g/150 g serving). The median protein level for flavored dairy yogurts was 17% higher at 4.2 g/100 g (or 6.3 g/150 g serving) [21].

Most of the protein in the non-dairy yogurts comes from legumes (soy and pea protein), and various seeds and nuts (but not coconut). With the exception of soy protein [22], these plant proteins have a biological value lower than that of animal proteins. A Dutch group recently reported the essential amino acid profile for pea protein to be quite similar to that of soy [23]. Recently, a French group pointed out that protein-rich plant foods, such as legumes, nuts and seeds, can achieve protein adequacy in adults consuming balanced plant-based diets [24].

The median level for calcium, in 57 dairy yogurts recently analyzed, was 10% of DV for calcium [19]. In our study, 45% of the yogurt alternatives overall were fortified with calcium to contain at least 10% DV/serving (Table 2). In a UK study, non-dairy plant-based yogurt alternatives had calcium levels of 115 mg/100 g and about 130 mg/100 g for dairy yogurts [21]. This corresponds to 172.5 and 195 mg calcium for a typical 150 g serving of yogurt, or about 15% DV for calcium. Predominantly, the yogurt alternatives based upon almond, coconut, oats, soy and a legume-blend are more likely to have adequate calcium fortification (Table 3). About one-half of the almond-based, coconut-based and legume-blend yogurts were fortified with calcium, while two thirds of the oat-based and 100% of the soy-based yogurt alternatives had calcium fortification. Almond- and oat-based yogurts had a median of 10% DV calcium/serving while soy had 15% DV and coconut/legume blends had a median of 20% DV of calcium/serving. Overall, any kind of fortification is limited to only one in three brands scattered amongst the various bases.

Four different calcium salts were used in the fortification of the yogurt alternatives, with tricalcium phosphate (TCP) and calcium citrate being the most commonly used, followed by calcium lactate and calcium carbonate. Most calcium salts used to fortify foods and beverages exhibit a bioavailability similar to that of milk calcium [25], which has a fractional absorption of about 30%. The absorbability of calcium varies depending upon the food matrix, including the pH and the presence of stabilizers [25,26]. Calcium carbonate absorption is very similar to milk calcium, while calcium lactate and calcium citrate tends to be a little better absorbed; TCP is absorbed less well than milk calcium [25].

Fortification of the non-dairy yogurt alternatives with vitamins D and B12 was not a common feature. It was less commonly observed than calcium fortification. A closer look at the nutritional adequacy of the yogurt alternatives (Table 2) found that only about one in five had vitamin D (17.8%) and vitamin B12 (21.7%) levels of fortification that reached at least at 10% DV level. While dairy yogurts are not commonly fortified with vitamin D, we found 30.9% of the non-dairy yogurts were fortified with vitamin D (Table 5). Most commonly it was the yogurts based on soy, oats, coconut or the legume blends that were vitamin D fortified (Table 3). The median level of vitamin D for our yogurt alternatives was 10% DV/serving (Table 1) which is the typical level of fortification for the dairy yogurts that are fortified. The use of vitamin D-fortified yogurts, compared to plain yogurt, has been found to improve one’s vitamin D status along with lower blood lipids and blood glucose levels [27,28]. Non-dairy yogurts are typically fortified with vitamin D2 rather than vitamin D3 since the latter is normally derived from animal sources, and unacceptable to vegans. However, vitamin D_2_ is equally as effective as vitamin D_3_ in maintaining 25-hydroxyvitamin D levels [29]. Coconut-, and oat-based, and legume-blend yogurt alternatives were the only products fortified with vitamin B12 (Table 3). These products were most commonly fortified at a level of 25–50% of DV/serving. Regular dairy yogurts contain about 1 µg of vitamin B12/serving (about 40% DV). Overall, only one in five non-dairy products (21.7%) had B12 fortification, while less than one-third (30.9%) had vitamin D fortification, and the fortification was brand-specific rather than dependent upon the type of yogurt. Of the 34 brands we analyzed, only 11 brands had calcium fortification while only 6 brands had both vitamin D and B12 fortification. When only the fortified non-dairy yogurt alternatives were analyzed, the calcium, vitamin D and B12 levels were more like those of regular dairy products. In this case, the median values for calcium, vitamin D and B12 were 15% DV, 10% DV, and 40% DV, respectively (Table 5).

### 4.1. Healthy Profile

The health profile of a product is typically assessed by the levels of salt (sodium), sugar and fat/saturated fat in the product, since these nutrients in excess are known to have negative health effects [30,31,32,33,34]. Consumers, for health reasons, are often concerned about the level of sodium, saturated fat and sugars in their food. A large majority of products (93.2%) had low levels of sodium. Most of the product bases were low in saturated fat except for those containing coconut. Very few products were high fat (only 8% had more than 20% of DV/serving), while more than one-half (53%) had high levels of sugar (at least 10 g sugar/serving). Only about 15% had no more than 5 g of sugar/serving (10% DV). The yogurt alternatives that are based on oats, seeds, or the coconut blends had lower levels of sugar, while those based upon oats, cashews, seeds, and the legume-blend had a lower content of sodium (Table 1). The yogurt alternatives based on oats, seeds, soy and the legume-blend had lower levels of fat and calories/serving. Soy-, pea-, almond-, coconut-, and cashew-based yogurts were the varieties most likely to contain higher levels of sugar and had median sugar contents of 10–16 g/serving. This compares with the 17 g/150 g serving for flavored yogurts analyzed from three different countries [35]. Coconut- and almond-based yogurts showed the highest median levels of fat, while soy-, oat-, seed-based, and legume blend yogurts had the lowest fat levels. The yogurts based on oats, seeds, and a legume blend contained the fewest calories/serving, while those based on almonds, coconut, and pea protein were the highest in calories/serving (Table 1). Furthermore, about one-half of the coconut-based yogurts also contained greater than 150 calories (Table 3). Only 9% of the yogurt alternatives contained over 190 calories/serving and were largely 3 brands. While three-quarters of these products were coconut-based, the balance were mix-ins with special toppings.

Over one-half (57%) of the yogurts studied had at least 1.5 g fiber/serving (Table 2) while the median level was 2 g/serving. The fibers added to the yogurt alternatives were mostly pectin, locust bean gum, and inulin. These water-soluble fibers, used as stabilizers and thickening agents in the yogurt alternatives, have properties that influence glycemia and hypercholesterolemia [36,37,38]. Inulin, which is added to 13% of the plant-based yogurts, is derived from either chicory root or agave. Inulin-type fructans act as prebiotics, since they have a beneficial effect on the human gut microbiota [39]. The gut bacteria convert inulin and other prebiotics into short-chain fatty acids, which nourish colon cells. Prebiotic fibers are considered a healthy addition since they improve digestive health and may also enhance immune function [36]. Some evidence suggests that prebiotics, as well as probiotics, may be beneficial in the prevention and treatment of colon cancer [40].

Plant-based yogurt alternatives typically contain a variety of live active cultures, similar to those used in dairy yogurts. Some of the products even advertise that they contain probiotic bacteria, as a part of their health messaging. In our study, the yogurt alternatives contained, on average, 4–6 probiotic live cultures. Common cultures that are added to the yogurt alternatives include *Streptococcus thermophilus*, *Lactobacillus acidophilus, Bifidobacterium* spp., *L rhamnosus*, L. casei, *Lactobacillus delbrueckii,* and *L.Bulgaricus.* The viable bacteria and bioactive metabolites of fermentation have long been associated with improved gut health. Their impact on the gut microbiome, may also improve overall health of the individual and immune function [41,42,43]. While the viability of probiotic microorganisms may be more difficult to maintain in a non-dairy matrix [44], the probiotics and bioactive compounds present in fermented non-dairy products have been associated with improved overall intestinal health and immune function by modifying the gut microbiome [44]. This has enabled the manufacturers of plant-based yogurt alternatives containing active cultures to make similar health claims as made for the dairy-based yogurts.

### 4.2. Comparison to Beverages

When dietary options are available for a vegetarian/vegan to choose between a non-dairy plant-based beverage and a non-dairy yogurt alternative, it is important to know what nutritional advantages may exist for one over the other (Table 4). Both product type and base type had a significant effect on almost all nutritional components (Table S3). The yogurt alternatives have about twice as much fat, sugar and calories/serving and five times more saturated fat than one serving of the non-dairy beverages while the beverages have 4–5 times more sodium than the yogurt alternatives (Table 4). While the non-dairy yogurt alternatives provide more protein/serving, the non-dairy beverages had significantly greater calcium and vitamin D levels.

When the data were divided according to compositional type, marked differences were seen (Table 6). The almond- and seed-based yogurt alternatives had 4 times the protein level of the non-dairy beverage counterparts while the legume-based yogurt alternatives had lower protein levels than the legume-based non-dairy beverages. While all the non-dairy beverages had substantially higher levels of calcium fortification than the yogurt alternatives, the differences were huge in the case of pea-, and seed-based, and the legume-blend products (Table S4). The oat-based and almond-based beverages even had calcium levels 2.5–3 times greater than the similar yogurts. While all yogurt alternatives, except soy-based yogurts, recorded a median of zero level of vitamin D, the plant-based beverages recorded a median vitamin D level of 10–30% DV/serving, except for cashew-based beverages (0% DV). In the same manner, all the non-dairy yogurt alternatives recorded a median of zero vitamin B12, while the soy-, coconut-, pea-based beverages reported a 42.5–50% DV B12/serving. All other beverages reported a median of zero B12/serving. While the non-dairy yogurt alternatives generally had more calories/serving than the non-dairy beverages, the differences were most pronounced for almond-, cashew-, and coconut-based products. Except for oat-based products, all types of non-dairy yogurts had 6–12 g more sugar/serving than the non-dairy beverages. Overall, the yogurt alternatives are more nutrient dense foods and provide more calories and macronutrients than the plant-based beverages/serving. However, fortification is a different story, as seen in Table 5. The beverages overall are twice as often fortified with calcium, vitamins D and B12, and the median level of fortification is substantially greater for the beverages. While many of the non-dairy yogurt alternatives lack fortification (Table 5), the different bases that are fortified with calcium, vitamin D and B12 have similar levels to the corresponding plant-based beverages, or they have significantly less nutrient fortification than the beverages (Table S4).

## 5. Conclusions

Consumers often choose a non-dairy yogurt alternative as a substitute for dairy yogurt. Sweetened, flavored varieties are the most commonly available types. Non-dairy yogurt alternatives are formulated largely from coconut (32%) and almonds (25%), while a significant number (24%) are either formulated from soy or pea protein or have a blend that includes a legume. At least one-third of the yogurt alternatives have 5 g or more of protein/serving, while a small number (4%) have 10–11 g protein/serving. Those products based upon a legume have the higher protein levels. A majority of the non-dairy yogurt alternatives were not fortified. Only 45% of the plant-based yogurts had calcium levels fortified to at least 10% of DV, while only about one in five had adequate vitamin D and B12 fortification at the 10% DV level. Fortification is very brand-specific, with only one-third of the 34 brands being fortified. The products that are based on oats, soy, or a coconut-legume mix demonstrate the best fortification. In comparison, while most regular dairy yogurts are not fortified with vitamin D, they do naturally contain both calcium (10% DV) and vitamin B12 (40% DV). In addition, the non-dairy, plant-based beverages tended to be better fortified (twice as frequently and at higher levels) than the non-dairy yogurt alternatives. One-half of the yogurt alternatives had high sugar levels, while 93% were low in sodium. Except for the coconut-based products, the yogurts were not high in fat or saturated fat. Less than 10% of the products contained over 190 calories/serving and the majority of these were coconut-based. The median level of dietary fiber in the yogurt alternatives was 2 g per serving. The most common fibers used were pectin, locust bean gum, and inulin. These prebiotic fibers are known to provide a number of health benefits. The presence of active cultures, 4–6 cultures on average, are also known to benefit the gut microbiota and improve overall health.

The observations made from this cross-sectional study represent just a window in time. New products continue to appear on the market, and formulations are not static. Fortification changes do occur with time. In addition, we have noticed that the nutrition information online is not always the same as what is presently seen on the nutrition label on products in the supermarkets, especially with respect to the level of fortification. When the fortification level changes, the company web page may reflect this change before the new products appear on the shelf or older stock has fully moved through the supply line. When a discrepancy was observed, the nutrient values appearing on products from the supermarket were utilized.

Typically, the company will analyze the nutritional composition of their product, and quality control measures will ensure that the final commercial products match the information placed on the nutrition label. However, some products provide the actual mg or gm provided by one serving along with the % DV. In doing the calculations for vitamins and minerals, one will observe that in the USA, numbers for % DV are often rounded to the nearest 5%. Also, for macronutrients, such as sugars, fats, protein, and dietary fiber, numbers are rounded without any decimals present. These procedures stand in contrast to European practices, where rounding is less noticeable and decimals commonly appear on the nutrition label. This gives the opportunity for generating more precise results. However, when we are working in this US project, with large quantities of products, these errors will be largely averaged out or minimized so that the big picture and the trends should still be visible to the researcher.

While consumers may have a taste preference or get a price advantage for a particular non-dairy yogurt alternative, they should be cognizant of the nutritional values of the various choices available to them. Carefully reading nutrition labels will enable the consumer to select a product with a good nutritional profile and a lower content of sugar and saturated fat.

## Figures and Tables

**Table 1:** The median (Q1–Q3) values of calories and 10 nutrients of non-dairy yogurts/serving classified according to their bases.

Product Base (n)	Calories	Total Fat (g)	Sat. Fat. (g)	Sodium (mg)	Carbs. (g)	Fiber (g)	Sugar (g)	Protein (g)	Calcium (% DV *)	Vit. D (% DV)	Vit. B12 (% DV)
Almond (62)	150 (140–180) ^a^	9.5 (7–11) ^ab^	1 (0.5–1) ^a^	49 (10–80) ^a^	19 (13–23) ^a^	3 (2–3) ^abc^	12.5 (8–15.8) ^a^	4 (3–5) ^a^	10 (4–10) ^abc^	0 (0–3) ^a^	0 (0–0) ^ab^
Cashew (13)	140 (140–150) ^bcde^	7 (6–7) ^cd^	1.5 (1–1.5) ^ab^	10 (10–11) ^b^	20 (19–20) ^abc^	1 (1–1) ^de^	12 (12–13) ^abc^	3 (3–3) ^ab^	2 (2–2) ^de^	0 (0–0) ^ab^	0 (0–0) ^a^
Coconut (79)	160 (127.5–190) ^ab^	8 (5.5–12.5) ^ac^	7 (5–11.5) ^c^	30 (20–55 ^a^	17 (10–22) ^bc^	2 (0.6–2) ^a^	10 (5.5–15) ^ab^	1 (0.3–1.5) ^c^	6 (0–25) ^a^	0 (0–10) ^c^	0 (0–25) ^b^
Oats (20)	120 (117.5–130) ^cf^	3.5 (3–4.8) ^f^	2.5 (1.8–2.5) ^b^	20 (10–25) ^b^	19 (19–20) ^ab^	1 (1–2) ^abf^	9 (7–9) ^cd^	3 (3–3) ^b^	10 (2–10) ^abf^	0 (0–10) ^c^	0 (0–10) ^bd^
Pea (5)	160 (160–160) ^abde^	6 (6–6) ^cdf^	0.5 (0.5–0.5) ^ad^	40 (40–40) ^ad^	19 (19–20) ^abc^	0 (0–0) ^e^	15 (15–15) ^ae^	6 (6–6) ^d^	0 (0–0) ^e^	0 (0–0) ^ab^	0 (0–0) ^ab^
Seeds ^1^ (7)	130 (130–130) ^cef^	4 (4–7) ^df^	0.5 (0.5–1) ^abd^	25 (0–26) ^bc^	14 (14–15) ^cd^	3 (3–3) ^def^	8 (8–9) ^bcd^	8 (5–8) ^d^	2 (2–2) ^def^	0 (0–0) ^ab^	0 (0–0) ^a^
Soy (11)	130 (130–145) ^cde^	3 (2.5–3.5) ^ef^	0.5 (0–0.5) ^d^	65 (57.5–87.5) ^d^	20 (18.5–23.5) ^a^	2 (1–2) ^c^	16 (12.5–20) ^e^	6 (6–6) ^d^	15 (8–15) ^c^	10 (0–10) ^ab^	0 (0–0) ^cd^
Coconut + legume ^2^ (23)	150 (140–180) ^abd^	7 (6.5–9) ^acd^	6 (6–7) ^c^	25 (15–57.5) ^ac^	14 (13–19.5) ^bc^	1 (0–1) ^bc^	9 (8–9.5) ^bcd^	6 (4–8) ^d^	20 (5–20) ^bc^	10 (5–40) ^d^	40 (10–40) ^c^
Coconut + seeds/nuts ^3^ (9)	160 (150–170) ^ab^	11 (10–11) ^b^	7 (5–7) ^c^	65 (10–65) ^ad^	11 (9–12) ^d^	1 (1–1) ^de^	7 (7–7) ^d^	2 (2–6) ^ab^	2 (2–2) ^de^	0 (0–0) ^ab^	0 (0–0) ^a^
Legume blend ^4^ (20)	110 (100–122.5) ^f^	1.5 (1.5–3.2) ^e^	0.5 (0–0.6) ^d^	5 (0–25) ^b^	18 (18–21) ^abc^	2 (1–2) ^df^	9.5 (8–12.25) ^abc^	6 (5–6) ^d^	2 (1.5–4) ^df^	0 (0–0) ^bc^	0 (0–0) ^ab^
Total (249)	150 (120–170)	7 (4–10)	2.5 (1–6)	25 (10–65)	19 (12–21)	2 (1–3)	10 (7–14)	3 (1–5)	10 (2–15)	10 (0–10)	20 (0–40)

^1^ hemp (*n* = 4), pumpkin seeds (*n* = 3). ^2^ coconut with pea protein. ^3^ coconut with pili nut (*n* = 6), coconut with cashews and watermelon and pumpkin seeds (*n* = 3). ^4^ oats with pea protein and/or fava bean (*n* = 16), almond with fava bean (*n* = 4). Kruskal–Wallis non-parametric tests for independent samples with multiple pairwise comparisons were used for each nutrient to perform comparisons among base types. Different lowercase letters (a,b,c, etc.) in the same column indicate significant differences among yogurt bases. * DV is the daily value. *P* < 0.05 is considered statistically significant.

**Table 2:** Percentage of 249 non-dairy yogurt alternatives meeting or exceeding suggested guideline per serving.

At least	
5 g protein	32.5
10% DV calcium	45.4
10% DV vitamin D	17.8
10% DV vitamin B12	21.7
1.5 g dietary fiber	57.0
No more than	
5 g sugar (10% DV)	15.3
1 g saturated fat (5% DV)	55.0
115 mg sodium (5% DV)	93.2
High levels	
More than 150 calories	40.6
10 g or more of sugar (20% DV)	53.0
4 g or more saturated fat (20% DV)	39.8
More than 15.5 g fat (20% DV)	7.6

**Table 3:** The percentage of non-dairy yogurt alternatives meeting or exceeding a suggested guideline [as in Table 2] separated according to the yogurt base.

Product Base (n)	At least 5 g Protein	At least 10% DV Calcium	At least 10% DV vit D	At least 10% DV vit B12	At least 1.5 g fiber	No more than 115 mg Sodium	No more than 5 g Sugar	No more than 1 g satd. fat	10 g or more Sugar	4 g or more satd. fat	More than 150 Calories	More than 15.5 g fat
Almond (62)	42	56	8	0	95	90	15	81	71	2	48	3
Cashew (13)	0	0	0	0	0	100	15	46	77	0	23	0
Coconut (79)	0	47	27	32	61	86	23	0	53	84	54	22
Oats (20)	0	65	40	40	45	100	10	25	20	0	25	0
Pea (5)	100	0	0	0	0	100	20	100	80	0	80	0
Seeds ^1^ (7)	100	0	0	0	100	100	0	100	0	0	0	0
Soy (11)	100	64	64	0	64	100	9	100	91	0	0	0
Coconut + legume ^2^ (23)	52	74	74	74	4	100	9	0	26	100	48	0
Coconut + seeds/nuts ^3^ (9)	33	0	0	0	0	100	11	0	22	100	56	0
Legume blend ^4^ (20)	85	20	20	20	55	100	10	95	50	0	0	0

^1^ hemp (*n* = 4), pumpkin seeds (*n* = 3). ^2^ coconut with pea protein. ^3^ coconut with pili nut (*n* = 6), coconut with cashews and watermelon and pumpkin seeds (*n* = 3). ^4^ oats with pea protein and/or fava bean (*n* = 16), almond with fava bean (*n* = 4).

**Table 4:** Medians (Q1–Q3) for calories and nutrients in non-dairy yogurt alternatives versus non-dairy, plant-based multi-serve beverages/serving.

	Yogurts	Beverages
*n*	249	326
Calories	150 (120–170) ^a^	80 (60–120) ^b^
Fat (g)	7 (4–10) ^a^	4 (2.5–5) ^b^
Saturated fat (g)	2.5 (1–6) ^a^	0.5 (0–0.9) ^b^
Sodium (mg)	25 (10–65) ^a^	110 (90–150) ^b^
Carbohydrates (g)	19 (12–21) ^a^	8 (2–15) ^b^
Fiber (g)	2 (1–3) ^a^	1 (0–1) ^b^
Sugar (g)	10 (7–14) ^a^	5 (0.1–8) ^b^
Protein (g)	3 (1–5) ^a^	2 (1–4) ^b^
Calcium (% DV)	10 (2–15) ^a^	25 (6–30) ^b^
Vitamin D (% DV)	10 (0–10) ^a^	15 (0–25) ^b^
Vitamin B12 (% DV)	20 (0–40) ^a^	0 (0–48.8) ^b^

Different lowercase letters in the same row indicate significant differences between the values for the non-dairy yogurt and the plant-based beverages. *P* < 0.05 is considered statistically significant.

**Table 5:** Medians (Q1–Q3) of fortification levels of calcium, vitamin D and vitamin B12 (expressed as % DV) in fortified non-dairy yogurt alternatives and the fortified non-dairy, plant-based multi-serve beverages.

	Yogurt		Multi–Serve Beverages	
	N (%)	Median	N (%)	Median
Calcium–fortified	117 (47.0%)	15 (10–20) ^a^	239 (73.3%)	30 (25–35) ^b^
Vitamin D–fortified	77 (30.9%)	10 (10–25) ^a^	229 (70.2%)	20 (10–25) ^b^
Vitamin B12 fortified	54 (21.7%)	40 (25–47.5) ^a^	138 (42.3%)	50 (25–60) ^b^

Different lowercase letters in the same row indicate significant differences between the values for the non-dairy yogurt and the plant-based beverages. *P* < 0.05 is considered statistically significant.

**Table 6:** Comparison of nutrient content of non-dairy yogurts and plant-based non-dairy beverages by base. For each yogurt base, different lowercase letters in the same column indicate significant differences between the plant-based beverages and the non-dairy yogurt alternatives. *P* < 0.05 is considered statistically significant.

Product Base (n)	Calories	Total Fat (g)	Sat. Fat. (g)	Sodium (mg)	Carbs. (g)	Fiber (g)	Sugar (g)	Protein (g)	Calcium (% DV)	Vit. D (% DV)	Vit. B12 (% DV)
Almond											
Yogurt (62)	150 (140–180) ^a^	9.5 (7–11) ^a^	1 (0.5–1)	49 (10–80) ^a^	19 (13–23) ^a^	3 (2–3) ^a^	12.5 (8–15.8) ^a^	4 (3–5) ^a^	10 (4–10) ^a^	0 (0–3) ^a^	0 (0–0)
Beverage (83)	60(35–80) ^b^	3 (2.5–3.5) ^b^	0 (0–0)	150 (120–170) ^b^	3 (1.5–8.5) ^b^	1 (0–1) ^b^	1 (0–7) ^b^	1 (1–1) ^b^	30 (4–35) ^b^	15 (0–25) ^b^	0 (0–0)
Cashew											
Yogurt (13)	140 (140–150) ^a^	7 (6–7)	1.5 (1–1.5)	10 (10–11) ^a^	20 (19–20) ^a^	1 (1–1)	12 (12–13) ^a^	3 (3–3)	2 (2–2)	0 (0–0)	0 (0–0)
Beverage (10)	50 (45–80) ^b^	4 (2.9–4.4)	0.25 (0–1)	95 (87.5–121.3) ^b^	3 (1–7.8) ^b^	0 (0–0)	0 (0–1.8) ^b^	1 (0.4–1.8)	7 (2.5–10)	0 (0–10)	0 (0–0)
Coconut											
Yogurt (79)	160 (127.5–190) ^a^	8 (5.5–12.5) ^a^	7 (5–11.5) ^a^	30 (20–55)	17 (10–22) ^a^	2 (0.6–2) ^a^	10 (5.5–15) ^a^	1 (0–1.5)	6 (0–25)	0 (0–10) ^a^	0 (0–25) ^a^
Beverage (24)	60 (50–80) ^b^	5 (4.5–5) ^b^	4.3 (4–5) ^b^	62.5 (30–113.8)	3 (1–7.3) ^b^	0 (0–0) ^b^	1 (0–7) ^b^	0 (0–0.5)	10 (10–30)	10 (10–25) ^b^	42.5 (18.8–50) ^b^
Oats											
Yogurt (20)	120 (117.5–130)	3.5 (3–4.8)	2.5 (1.8–2.5)	20 (10–25) ^a^	19 (19–20)	1 (1–2)	9 (7–9)	3 (3–3)	10 (2–10) ^a^	0 (0–10)	0 (0–10)
Beverage (62)	120 (90–130)	4.3 (2–5)	0.5 (0–0.5)	105 (100–120) ^b^	16 (14–21)	2 (1–2)	6.5 (4–9)	2 (1–3)	25 (2–25) ^b^	10 (0–20)	0 (0–40)
Pea											
Yogurt (5)	160 (160–160)	6 (6–6)	0.5 (0.5–0.5)	40 (40–40) ^a^	19 (19–20)	0 (0–0)	15 (15–15)	6 (6–6)	0 (0–0) ^a^	0 (0–0) ^a^	0 (0–0) ^a^
Beverage (14)	105 (90–136.3)	4.5 (4.5–5.8)	0.5 (0.5–0.7)	125 (96.3–160) ^b^	7 (6–12.8)	0 (0–1)	5.5 (3–11.8)	8 (4.3–8)	35 (25–35) ^b^	30 (25–30) ^b^	45 (35–100) ^b^
Seeds											
Yogurt (7)	130 (130–130)	4 (4–7)	0.5 (0.5–1)	25 (0–26) ^a^	14 (14–15)	3 (3–3) ^a^	8 (8–9)	8 (5–8) ^a^	2 (2–2) ^a^	0 (0–0)	0 (0–0)
Beverage (25)	60 (50–80)	4.5 (2.5–6)	0.1 (0–0.5)	100 (90–125) ^b^	4 (1–10)	1 (0–2) ^b^	0 (0–7)	2 (2–3) ^b^	25 (20–30) ^b^	10 (3–10)	0 (0–25)
Soy											
Yogurt (11)	130 (130–145)	3 (2.5–3.5)	0.5 (0–0.5)	65 (57.5–87.5)	20 (18.5–23.5) ^a^	2 (1–2)	16 (12.5–20) ^a^	6 (6–6) ^a^	15 (8–15)	10 (0–10) ^a^	0 (0–0) ^a^
Beverage (44)	100 (90–130)	4 (3.9–4.6)	0.5 (0.5–0.6)	95 (85–125)	8.5 (4–11.3) ^b^	1 (1–2)	6 (2–9) ^b^	7 (7–9) ^b^	25 (23.8–30)	15 (15–25) ^b^	50 (0–75) ^b^
Legume blend^1^											
Yogurt (20)	110 (100–122.5)	1.5 (1.5–3.1)	0.5 (0–0.6)	5 (0–25) ^a^	18 (18–21) ^a^	2 (1–2)	9.5 (8–12.3)	6 (5–6) ^a^	2 (1.5–4) ^a^	0 (0–0) ^a^	0 (0–0)
Beverage (18)	115 (92.5–140)	5 (3.1–6.5)	0.5 (0–0.5)	160 (150–190) ^b^	9.5 (2–11.8) ^b^	0.3 (0.2–1)	3 (0–10.8)	8 (8–10) ^b^	30 (25–30) ^b^	12.5 (10–48.8) ^b^	0 (0–37.5)

^1^ a product with pea protein, soy, or fava bean.

## Supplementary Materials

The following are available online at https://www.mdpi.com/article/10.3390/nu13114069/s1, Table S1: Nutrient profile of a leading brand of 2% dairy yogurt; Table S2: Nutritional analysis of dairy and non-dairy plant-based yogurt alternatives; Table S3. The effects of product type (non-dairy yogurts versus non-dairy beverage) and base type; Table S4: Median (Q1–Q3) of the fortification levels of Calcium, Vitamin D and B12 (expressed as % DV) of non-dairy yogurt alternatives and non-dairy, plant-based beverages.
