# Supplementary material for: Nutritional Content and Health Profile of Non-Dairy Plant-Based Yogurt Alternatives
Source: Nutrients. 2021 Nov 14;13(11):4069. doi: 10.3390/nu13114069 (PMC8619131; doi:10.3390/nu13114069)
Supplement: Supplementary file 1 [file nutrients-13-04069-s001.zip › nutrients-1396896-supplementary.pdf]

Table S1. Nutrient profile of a leading brand of 2% dairy yogurt[1].

Calories	150
Fat (g)	3
Saturated fat (g)	2
Sodium (mg)	40
Carbohydrates (g)	16
Fiber (g)	0
Sugars (g)	13
Protein (g)	14
Calcium (% DV)	8
Vitamin D (% DV)	0
Vitamin B12 (% DV)	n/a

[1]Data from nutrition label of Tillamook yogurt. n/a = not available

Table S2. Median (Q1–Q3) values of Selected Nutrients[1] of Dairy and Non-dairy plant-based yogurt alternatives.

	**Dairy yogurts**	**Non-dairy yogurt alternatives**	
n	57	25	p-value
Calories (kcal)	130 (110–150)[a]	145 (130–170)[b]	0.004
Saturated fat (g)	1.5 (1–2.5)	1 (0.5–2.87)	0.45
Protein (g)	12 (11–15)[a]	4.5 (3–6)[b]	<0.001
Calcium (% DV)	10 (10–15)[a]	10 (4–10)[b]	0.007

[1]Analysis of data reported in ref [26].

Different lowercase letters in the same row indicates significant differences between dairy and non-dairy alternatives. P<0.05 is considered statistically significant.

Table S3. The effects of product type (non-dairy yogurts versus non-dairy beverage) and base type (almond, cashew, etc.).

**Factor**	df	F-value	p-value
Calories			
**Product**	**1**	**65.65**	**7.93E-14**
**Base**	**8**	**6.82**	**8.25E-08**
**Product x Base**	**2**	**9.94**	**8.07E-05**
Fat			
**Product**	**1**	**16.65**	**6.75E-05**
**Base**	**8**	**4.88**	**1.84E-05**
Product x Base	2	2.99	5.26E-02
Saturated Fat			
**Product**	**1**	**472.02**	**<2E-16**
**Base**	**8**	**21.93**	**<2E-16**
**Product x Base**	**2**	**17.93**	**7.91E-08**
Sodium			
**Product**	**1**	**140.4**	**<2E-16**
**Base**	**8**	**3.6**	**6.58E-04**
**Product x Base**	**2**	**13.85**	**2.55E-06**
Total Carbohydrates			
**Product**	**1**	**100.12**	**<2E-16**
**Base**	**8**	**10.34**	**7.25E-12**
**Product x Base**	**2**	**12.69**	**6.99E-06**
Fiber			
**Product**	**1**	**4.61**	**3.30E-02**
**Base**	**8**	**6.66**	**1.29E-07**
**Product x Base**	**2**	**5.48**	**5.00E-03**
Sugar			
**Product**	**1**	**48.6**	**5.75E-11**
**Base**	**8**	**2.32**	**2.20E-02**
**Product x Base**	**2**	**5.75**	**4.00E-03**
Protein			
**Product**	**1**	**20.59**	**1.04E-05**
**Base**	**8**	**62.83**	**<2E-16**
**Product x Base**	**2**	**10**	**7.65E-05**

Calcium			
**Product**	**1**	**17.81**	**3.86E-05**
**Base**	**8**	**6.86**	**7.35E-08**
**Product x Base**	**2**	**11.31**	**2.37E-05**
Vitamin D			
Product	1	0.15	6.99E-01
**Base**	**8**	**6.36**	**2.90E-07**
**Product x Base**	**2**	**15**	**9.46E-07**
Vitamin B12			
**Product**	**1**	**20.07**	**1.32E-05**
**Base**	**8**	**7.55**	**1.12E-08**
Product x Base	2	0.72	4.88E-01

Analysis was conducted using a two-way analysis of variance for each nutrient. Significant factors ($P<0.05$) are in bold

Table S4. Median (Q1-Q3) of the fortification levels of Calcium, Vitamin D and B12 (expressed as % DV) of non-dairy yogurt alternatives and non-dairy, plant-based multi-serve beverages.

	**Calcium**				**Vitamin D**				**Vitamin B12**			
	**Yogurt**		**Beverage**		**Yogurt**		**Beverage**		**Yogurt**		**Beverage**	
	n	median (Q1–Q3)	n	median (Q1–Q3)	n	median (Q1–Q3)	n	median (Q1–Q3)	n	median (Q1–Q3)	n	median (Q1–Q3)
Almond	35	10 (10–11)[a]	53	30 (30–35)[b]	16	6 (6–10)[a]	58	25 (15–25)[b]	0		13	25 (25–35)
Cashew	0		5	10 (10–45)	0		4	17.5 (10–25)	0		2	120 (120–120)
Coconut	37	25 (15–30)	19	20 (10–32.5)	25	10 (10–25)	20	12.5 (10–25)	25	40 (25–50)	14	50 (35–120)
Oats	13	10 (10–10)[a]	44	25 (20–25)[b]	8	10 (10–10)	38	20 (11.25–20)	8	30 (10–50)	29	40 (25–50)
Pea	0		14	35 (25–35)	0		14	30 (25–30)	0		14	45 (35–100)
Seeds	0		20	30 (20–30)	0		19	10 (10–25)	0		11	25 (25–50)
Soy	11	15 (8–15)[a]	39	25 (25–30)[b]	7	10 (10–10)	35	25 (15–25)	0		32	50 (50–120)
Legume blend	21	20 (17–20)[a]	18	30 (25–30)[b]	21	35 (10–40)	18	12.5 (10–43.8)	21	40 (30–40)	5	60 (60–60)
Others	0		27	30 (25–35)	0		23	25 (15–25)	0		18	50 (25–50)
Total	117	15 (10–20)[a]	239	30 (25–35)[b]	77	10 (10–25)[a]	229	20 (10–25)[b]	54	40 (25–47.5)[a]	138	50 (25–60)[b]

Different lowercase letters indicate significant differences within a nutrient between non-dairy yogurt alternatives and non-dairy, plant-based multi-serve beverages. $P<0.05$ is considered statistically significant.
